# Improvement in Adverse Drug Reaction (ADR) Knowledge: A Pre- and Post-video Intervention Study Among Doctors

**DOI:** 10.7759/cureus.67622

**Published:** 2024-08-23

**Authors:** Krima S Patel, Shivam Patel, Shreya Patel, Digantkumar Patel, Devang A Rana, Viray Shah, Supriya Malhotra

**Affiliations:** 1 Department of Medicine, Smt. Nathiba Hargovandas Lakhmichand (NHL) Municipal Medical College, Ahmedabad, IND; 2 Department of Pharmacology, Narendra Modi Medical College, Ahmedabad, IND; 3 Department of Medicine, Springfield Memorial Hospital, Springfield, USA; 4 Department of Pharmacology, Smt. Nathiba Hargovandas Lakhmichand (NHL) Municipal Medical College, Ahmedabad, IND; 5 Department of Hospital Medicine, MedStar Good Samaritan Hospital, Baltimore, USA

**Keywords:** doctors, reporting, awareness, intervention, pharmacovigilance, adverse drug reaction

## Abstract

Background

Adverse drug reactions (ADRs) represent a significant public health concern, contributing to mortality, morbidity, and healthcare costs worldwide. Healthcare practitioners especially doctors play a vital role in identifying and reporting ADR. This study investigates the prevalence of knowledge regarding ADR among doctors and enhances it with educational intervention. It also explores the association between demographic factors and baseline ADR awareness.

Methods

A prospective cross-sectional interventional study was conducted among doctors in Ahmedabad, India, to evaluate their knowledge of ADR reporting and the effectiveness of an educational video intervention. Pre- and post-intervention questionnaires were administered to assess knowledge improvement. Statistical analysis, including paired t-tests and chi-square tests, was performed to evaluate the intervention's impact and explore associations between demographic factors and ADR awareness.

Results

Analysis of pre- and post-intervention questionnaires revealed a significant increase in correct response rates post-intervention, indicating the effectiveness of the educational video intervention. Demographic factors, particularly age, were associated with ADR awareness. Following the intervention, participants demonstrated an improved understanding of ADR definitions, WHO causality assessment, reporting mechanisms, and challenges faced by pharmacovigilance programs. All participants found the video helpful and expressed intent to share their knowledge post-intervention.

Conclusion

The results of the study suggest that educational video intervention can serve as an effective tool for understanding ADR concepts and pharmacovigilance practices. Moreover, the association of demographic factors, particularly age, with ADR awareness further emphasizes the importance of educational interventions in addressing specific population needs.

## Introduction

According to the World Health Organization (WHO), an adverse drug reaction (ADR) is defined as “a noxious, unintended, and undesirable effect that occurs as a result of a dose normally used in man for diagnosis, prophylaxis, treatment of disease, or modification of physiological function” [1]. Adverse drug reactions result in diminished quality of life, increased physician visits, hospitalizations, and even death. In addition, they result in increased healthcare costs worldwide. The impact and management of ADRs are complex, and in the USA, they may cost up to 30.1 billion dollars annually [2]. ADRs may increase costs due to increased hospitalization, prolongation of hospital stay, and additional clinical investigations in more serious cases [2]. 

ADRs impose significant burdens on hospitals by prolonging patient stays and increasing admission rates [3]. Prevalence studies in various settings showed that approximately 5%-35% of many preventable hospital admissions are due to ADRs, with a huge financial burden [4]. At least one ADR has been reported to occur in 10%-20% of hospitalized patients [5]. According to a recent meta-analysis, 1.6% of in-patients had preventable ADRs, and 45% of the ADRs were assessed as preventable [6].

It is the fourth- to sixth-leading cause of mortality in the United States of America [7]. In a hospital setting, ADRs can happen due to the severity of the illness, the use of multiple drugs, drug interactions, or potential negligence [8]. This should lead to a heightened awareness of ADRs, increased reporting of ADRs, and increased opportunities for drug review, drug selection, and prescribing practices affecting patient outcomes [9].

The ADR reporting rate is below 1% in India compared to the worldwide rate of 6%-10% [10]. A systematic review of the determinants of underreporting of ADRs identified the following factors: ignorance, lack of interest, unavailability of reporting forms, lack of time, and not being sure whether the report will make any difference [11].

The study of ADRs is the concern of the field known as pharmacovigilance. According to the WHO, pharmacovigilance (PV) is defined as the science and activities relating to the detection, assessment, understanding, and prevention of adverse effects or any other drug-related problem [12]. To transform the pharmacovigilance activity into practices for enhancing the safety of patients, more ADR monitoring centers are being set up across the country under the Pharmacovigilance Program of India (PvPI) [13]. Despite the efforts of the Drug Controller General of India (DCGI) and the Indian Council of Medical Research (ICMR) in establishing ADR monitoring centers in many hospitals in the major cities of India and the presence of a large number of tertiary care facilities, pharmacovigilance is still in its infancy in India [14].

Healthcare professionals, especially doctors, play a vital role in reporting ADR. Enhancing doctors’ awareness and understanding of ADR reporting is essential for improving pharmacovigilance practices and patient safety. This study aims to explore the current state of knowledge regarding ADR reporting among doctors, identify barriers to facilitate reporting, and elucidate the pivotal role of doctors.

Objectives

To evaluate the knowledge regarding ADR reporting among doctors practicing in different settings. To create awareness regarding the current reporting system for adverse drug reactions.

## Materials and methods

Study design

Prospective cross-sectional interventional: The study utilized pre- and post-intervention questionnaires to evaluate the efficacy of a video intervention in enhancing doctors' knowledge of ADRs. Additionally, it analyzed the relationship between intervention impact and demographic variables, including age, gender, and occupation, categorized as private practitioners, hospitalists, and residents. The data was analyzed using Microsoft Excel v16.75.2 (Microsoft Corp., Redmond, USA), a paired t-test for the intervention, and a chi-square test for demographic associations.

Study duration and location

The study was conducted from January 2024 to February 2024 and was carried out across public and private healthcare facilities situated in Ahmedabad, Gujarat, India.

Study participants

The study participants included practitioners such as physicians and residents working at Sardar Vallabhbhai Patel Hospital, Ahmedabad, Gujarat, India, as well as physicians working at private clinics and hospitals. Participants were recruited randomly. Before the commencement of the study, informed consent was obtained from each participant. The study began after the permission of the study protocol by the Institutional Review Board and confidentiality of all data was maintained.

Development and validation of questionnaires

The questionnaire included questions based on knowledge about ADR reporting and pharmacovigilance. Manuscripts and published papers outlining similar research were also studied [15]. The questionnaire was structured to collect demographic details of doctors and to evaluate the knowledge of ADR reporting, the type of ADRs to be reported, who can report ADRs, their seriousness criteria, and challenges faced by the National Pharmacovigilance Centre. Information about the scales used to analyze ADRs and the WHO online database to report ADRs were also addressed in the questionnaire. Both questionnaires were validated by Cronbach’s alpha = 0.754. Both questionnaires were designed in the English language.

Pre-video questionnaire

Before the video intervention, pre-video questionnaires were completed by participants to assess their existing knowledge regarding ADRs. The questionnaires were circulated to the participants via Google Forms (Google LLC, Mountain View, USA). 

Video intervention

The intervention consisted of a three-minute educational video generated with the help of artificial intelligence (AI). It was designed to enhance knowledge regarding ADRs and the importance of ADR reporting. The video covered key aspects, including ADR identification, a reporting mechanism, current challenges, and potential implications of ADRs in clinical practices. Participants were provided access to the video (see Video 1 in the Appendices). 

Post-video questionnaire

After the video, participants were asked to complete post-video questionnaires to assess their knowledge acquisition and understanding regarding ADR reporting. Post-video questionnaires were circulated via Google Forms. 

## Results

A total of 120 doctors were invited to participate in the study. Out of these, 104 doctors filled out the pre-video questionnaire and, from them, 98 doctors followed up on the video intervention and filled out the post-video questionnaire. Demographic details of the study participants are shown in Table 1.

**Table 1:** Demographic details of the study participants

Characteristics	Number (Percentage) n=104	Number (Percentage) n=98
	Pre-video questionnaire study participants	Post-video questionnaire study participants
Gender		
Male	63 (60.55)	59 (60.2)
Female	41 (39.45)	39 (39.8)
Age		
<30 years	60 (57.6)	57 (58.16)
>30 years	44 (43.4)	41 (43)
Occupation		
Residents	34 (32.6)	32 (32.6)
Hospitalist physicians	41 (39.4)	38 (38.7)
Private practice physicians	29 (27.8)	28 (28.5)

Pre-video questionnaire

The frequency of study participants according to the answers in the pre-video questionnaire is shown in Table 2.

**Table 2:** Frequency of study participants according to the answers in the pre-video questionnaire ADR: Adverse drug reaction; WHO: World Health Organisation; ICSR: Individual case safety reports; ALDEN: Algorithm of drug causality for epidermal necrolysis; PvPI: Pharmacovigilance Program of India

Questions	Options	Associated Frequency
Aware of the term ADR?	No	5 (4.8)
	Yes	99 (95.2)
Define ADR	Noxious and unintended response to drug and occurs at doses normally used in man or animal for prophylaxis, diagnosis, or therapy of disease	34 (32.7)
	Noxious and unintended response to drug and occurs at doses normally used in man for prophylaxis, diagnosis, and therapy of disease	44 (42.3)
	Any adverse reaction identified in regulatory documents occurring within the expected frequency	10 (9.6)
	Any untoward medical occurrence that may present during treatment with medicine but does not necessarily have a casual relationship with this treatment	16 (15.4)
Which of the following is most commonly used to establish the ADR causality?	Hartwig scale	11 (10.6)
	WHO causality assessment	24 (23.1)
	ALDEN scale	2 (1.9)
	Hartwig scale, WHO causality assessment, ALDEN scale	21 (20.2)
	Did not know	46 (44.2)
Are you aware of any drug that has been banned in the world due to ADR?	No	34 (32.7)
			Yes	70 (67.3)
			Do you know how to report ADR?	No	53 (51)
	Yes	51 (49)			
Who can report ADRs?	Doctors	17 (16.3)	
			Both doctors and nurses	16 (15.4)
	Doctors, nurses, pharmacists, marketing holders	52 (50)
	Did not know	19 (18.3)
	Do you know regarding the ADR reporting form?	No	55 (52.9)
Yes		49 (47.1)
How many sections are present in the ADR reporting form to validate ICSR (Individual case safety report?	2	8 (7.7)
	3	11 (10.6)
	4	9 (8.7)
	Did not know	76 (73.1)
What type of ADR should be reported?	Known ADR	10 (9.6 )
			Unknown ADR	1 (1)
	Serious/non-serious ADR	4 (3.8)
	Known ADR, unknown ADR, serious/non-serious ADR	64 (61.5)
	Did not know	25 (24)
	ADR seriousness criteria include:	Death	1 (1)
Life-threatening		5 (4.8)
Required intervention to prevent permanent damage		1 (1)
Disability, hospitalization, death, life-threatening, congenital anomaly, required intervention to prevent permanent damage		76 (73.1)
Did not know		21 (20.2)
Do you know about PvPl?	No	49 (47.1)
	Yes	55 (52.9)
Which of the following is the WHO online database for reporting ADRs?	ADR advisory committee	16 (15.4)
	Medsafe	3 (2.9)
	VigiBase	17 (16.3)
	Did not know	68 (65.4)
Have you shared information about ADRs with anyone?	No	65 (62.5)
	Yes	39 (37.5)

Post-video questionnaire

The frequency of study participants according to the answers in the post-video questionnaire is shown in Table 3.

**Table 3:** Frequency of study participants according to the answers in the post-video questionnaire ADR: Adverse drug reaction; WHO: World Health Organisation; ICSR: Individual case safety reports; ALDEN: Algorithm of drug causality for epidermal necrolysis; PvPI: Pharmacovigilance Program of India

Questions	Answers	Frequency
Aware of the term ADR?	Yes	98 (100)
Define ADR	Noxious and unintended response to drug and occurs at doses normally used in man or animal for prophylaxis, diagnosis, or therapy of the disease	4 (4.1)
	Noxious and unintended response to drug and occurs at doses normally used in man for prophylaxis, diagnosis, or therapy of disease	93 (94.9)
	Any untoward medical occurrence that may present during treatment with medicine but does not necessarily have a casual relationship with this treatment	1 (1)
Which of the following is most commonly used to establish the ADR causality?	Hartwig scale	2 (2)
			WHO causality assessment	86 (87.8)
	Hartwig scale, WHO causality assessment, ALDEN scale	10 (10.2)
	Do you know how to report ADR?	Yes	98 (100)
Who can report ADRs?	Both doctors and nurses	5 (5.1)
	Doctors, nurses, pharmacists, marketing holders	93 (94.9)
Do you know regarding the ADR reporting form?	Yes	98 (100)
How many sections are present in the ADR reporting form to validate ICSR (Individual case safety report)?	2	4 (4.1)
	3	18 (18.4)
	4	72 (73.5)
	Did not know	4 (4.1)
What type of ADR should be reported?	Known ADR, unknown ADR, serious/non-serious ADR	98 (100)
ADR seriousness criteria include:	Disability, hospitalisation, death, life-threatening, congenital anomaly, required intervention to prevent permanent damage	98 (100)
Do you know about PvPl?	No	1 (1)
	Yes	97 (99)
Which of the following is the WHO online database for reporting ADRs?	ADR advisory committee	19 (19.4%)
	VigiBase	79 (80.6)
Did you find the video helpful?	Yes	98 (100)
Will you share this knowledge regarding ADR reporting to the community?	Yes	98 (100)

**Figure 1:**
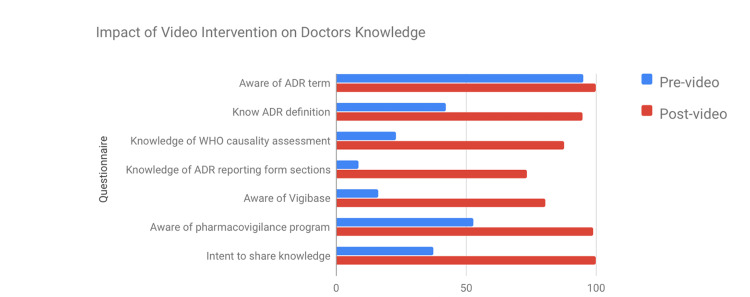
Knowledge improvement before and after intervention among doctors ADR: Adverse drug reaction

In the study with 104 participants, awareness of ADR was 95.2%, but understanding of its definition was only 42.3% pre-video. After the video, awareness of the definition increased to 94.9%. Knowledge of WHO causality assessment increased from 23.1% to 87.8%. Familiarity with the ADR reporting form increased from 47.1% to 100%. Understanding of its sections rose significantly from 8.7% to 73.5%. Knowledge of India's pharmacovigilance program increased from 52.9% to 99%, and awareness of its challenges rose from 42.3% to 98%. Knowledge about the WHO online database VigiBase increased from 16.3% to 80.6%. All 98 doctors found the video helpful and intended to share their knowledge post-intervention, while only 37.5% had shared it before (Figure 1).

Statistical analysis

The mean correct percentage (mean was derived from the correct percentages of each variable) for the pre-video and post-video questionnaires were compared, comprising 11 identical questions each, to 98 participants before and after the intervention. Participants who did not follow up were excluded from the analysis to ensure data integrity.

To evaluate participants' performance, we analyzed the correct percentage of responses for both pre-intervention and post-intervention questionnaires separately. This was achieved by dividing the total number of correct responses by 11. The mean correct percentage for the pre-intervention questionnaire was determined to be 38.37±22.3%, while for the post-intervention questionnaire, it significantly increased to 93.5±5.5%.

Using a paired t-test, we compared the mean correct percentages before and after the intervention, revealing a statistically significant difference with a p-value of p<0.01. These findings underscore the substantial impact of the intervention on enhancing participants' knowledge regarding the reporting of ADRs, as evidenced by the remarkable improvement in correct response rates following the intervention.

Furthermore, to explore the association between the prevalence of awareness and demographic factors such as gender, age, and occupation, we employed a chi-square test. This analysis, conducted using the pre-intervention questionnaire data from the 98 participants who followed up, is detailed in Table 4. Such insights into demographic influences can inform targeted interventions and strategies aimed at optimizing pharmacovigilance practices across diverse healthcare settings.

**Table 4:** Association of age, gender, and occupation with degree of awareness

	Unaware	Moderately Aware	Aware	P-value
Age				
⩽30	1	39	17	0.035
>30	5	30	6	
Gender				
Male	3	43	13	0.7
Female	3	26	10	
Occupation				
Hospital physician	1	25	12	0.3
Private practitioners	3	19	6	
Residents	2	25	5	

The association between these variables was significant (p=0.035). Younger participants were more likely than older participants to have overall awareness of reporting ADRs.

The results of this study demonstrate a significant improvement in the knowledge and awareness of ADRs among doctors following the implementation of educational video intervention. The results suggest that the video effectively conveyed key information related to ADR recognition, the importance of its reporting, mechanisms of reporting, its associated implications, and the challenges faced by PvPI.

## Discussion

The pre-intervention survey revealed a significant gap in ADR knowledge among participating doctors, consistent with previous research indicating suboptimal awareness and reporting practices. Our study classified the doctors as residents, private practice physicians, and hospital physicians. A similar study has been conducted in Gujarat, solely on the resident doctors from the clinical and para-clinical departments, in which 66% of the residents were unaware of PvPI [16]. The questionnaire framed for our study had statements in the options for the participants to choose from. There are reports where a scoring pattern has been used to frame the questionnaire options, ranging from one (strongly disagree) to five (strongly agree) [17,18]. The mode of educational intervention used in our study was a video presentation, whereas other studies have also reported using oral presentations, PowerPoint presentations, and interactive sessions [16,17,18]. Apart from the theoretical knowledge, the practical aspect is also handled via hands-on training on ADR documentation, such as filling out the ADR reporting form, etc. [16]. Following the video intervention, a notable improvement in ADR knowledge scores was observed among participants, indicating the effectiveness of the educational intervention. This underscores the need for targeted educational interventions to address knowledge deficits and improve ADR recognition and reporting. Some studies showcased the retention of information post-intervention, classified as retention data, such as a study conducted in Nepal that showed the participants' knowledge scores post six weeks of the intervention to understand the gain of knowledge among the participants [17]. 

The mean correct percentage of the video questionnaire was 32.4 among 98 participants. These findings emphasize the need for education and training initiatives in the healthcare setting to address specific deficiencies and ensure a comprehensive understanding of ADR-related concepts. Post-video questionnaires revealed the acquisition and retention of knowledge of health care practitioners after the video intervention. The mean correct percentage of the post-video questionnaire improved to 93.5 among 98 participants.

Findings from studies conducted in Ethiopia and Bhutan also found that doctors and nurses were less knowledgeable about ADR reporting than pharmacists [19,20].

As opposed to our study, in which there is no statistical significance between the awareness of ADR between males and females, [17,18] reported the improvement in knowledge scores of females as compared to males, whereas Jha et al. [17] reported a consistent performance in males with respect to the knowledge scores as compared to the females who didn’t have a statistically significant difference post six weeks of educational intervention. However, the overall improvement scores were higher in females. Our study showcases a significant difference in ADR awareness among people below 30, which is supported by other studies as well [17,18].

There are various reasons for underreporting of ADRs among healthcare practitioners (HCPs) such as lack of knowledge, time constraints, concerns about liability, attitudinal factors, confidentiality concerns, and lack of incentives. 

The use of incentives was also suggested by HCPs in recent studies conducted in Pakistan and China [21,22,23]. According to the study done in Iran, the main reasons for underreporting of ADR were lack of information from the patient, being too well known to report, not knowing how to report, uncertain association, and being unaware of the existence of a national ADR reporting system [15]. A study conducted by Ramesh and Parthasarathi [24] stated that doctors were less aware or lacked knowledge of national and international pharmacovigilance programs. 

Other causes of underreporting in the study held in Barcelona, Spain, were lack of time, most importantly, lack of information about the spontaneous reporting system, unavailability of yellow cards, doubt of ADR causality assessment, and lack of patient confidentiality [25]. Whereas various studies from developed countries like the United Kingdom and the United States have shown a higher rate of ADR reporting and relatively higher awareness and knowledge about pharmacovigilance among HCPs [26,27,28].

Strengths and limitations

Utilizing a classification system based on qualifications and practice types offers a valuable metric for assessing the current state of knowledge prevalence among HCPs. This approach enables targeted interventions to address specific knowledge gaps within distinct practitioner groups. Our study encountered constraints due to the inability to employ scoring-based questionnaires, which could have provided more granular insights into participants' comprehension levels. Additionally, we were unable to establish correlations between demographic data and awareness levels and the magnitude of improvement in ADR knowledge post-intervention, limiting the depth of our analysis. Another limitation could be that the doctors might not recall the salient features of the video if they were presented with a similar questionnaire in a few months' time.

## Conclusions

The observed outcomes corroborate prior research affirming the effectiveness of multimedia interventions, notably videos, in encouraging HCPs' grasp of pharmacovigilance principles and ADR management. It indicates that readily accessible and engaging educational materials can serve as potent tools, thereby empowering physicians to report ADR and share their knowledge with the community.

This finding holds significant implications, considering the pivotal role of doctors in pharmacovigilance activities and the importance of timely ADR identification. By conducting such innovative educational strategies, healthcare systems can foster a culture of heightened awareness towards medication safety. Moving forward, there is potential to employ the questionnaire and accompanying video as part of ongoing medical education initiatives for healthcare professionals. By integrating these resources, we can enhance sustained knowledge regarding pharmacovigilance and ADR management activities.

## Appendices

Pre-video questionnaire

Are you aware of the term ADR? Yes No

Define ADR A. Noxious and unintended response to drug and occurs at doses normally used in man or animal for prophylaxis, diagnosis, or therapy of disease B. Noxious and unintended response to drug and occurs at doses normally used in man for prophylaxis, diagnosis, and therapy of disease C. Any adverse reaction identified in regulatory documents occurring within the expected frequency D. Any untoward medical occurrence that may present during treatment with medicine but does not necessarily have a casual relationship with this treatment E. Do not know

Which of the following is most commonly used to establish the ADR causality? A. Hartwig scale B. WHO causality assessment C. ALDEN scale D. All of these E. Do not know

Are you aware of any drug that has been banned in the world due to ADR? Yes No

Do you know how to report ADR? Yes No

Who can report ADRs? A. Doctors B. Nurses C. Pharmacists D. Marketing holders E. Both A and B F. All of these G. Do not know Other:

Do you know regarding the ADR reporting form? Yes No

How many sections are present in the ADR reporting form to validate ICSR (individual case safety report)? A. 1 B. 2 C. 3 D. 4 E. Do not know

What type of ADR should be reported? A. Known ADR B. Unknown ADR C. Serious/non-serious ADR D. All of these E. Do not know

ADR seriousness criteria include: A. Disability B. Hospitalisation C. Death D. Life-threatening E. Congenital anomaly F. Required intervention to prevent permanent damage G. All of these H. Do not know

Do you know about PvPI? Yes No

Which of the following challenges occur for implementing PvPI? A. Political pressure B. Lack of trained personal C. Underreporting culture D. Inadequate communication E. All of these F. Do not know

Which of the following is the WHO online database for reporting ADRs? A. ADR advisory committee B. Medsafe C. VigiBase D. MedWatch E. Do not know

Have you shared information about ADRs with anyone? Yes No

Post-video questionnaire

Are you aware of the term ADR? Yes No

Define ADR A. Noxious and unintended response to drug and occurs at doses normally used in man or animal for prophylaxis, diagnosis, or therapy of the disease B. Noxious and unintended response to drug and occurs at doses normally used in man for prophylaxis, diagnosis, or therapy of disease C. Any adverse reaction identified in regulatory documents occurring within the expected frequency D. Any untoward medical occurrence that may present during treatment with medicine but does not necessarily have a casual relationship with this treatment E. Do not know

Which of the following is most commonly used to establish the ADR causality? A. Hartwig scale B. WHO causality assessment C. ALDEN score D. All of these E. Do not know

Do you know how to report ADR? Yes No

Who can report ADRs? A. Doctors B. Nurses C. Pharmacists D. Marketing holders E. Both A and B F. All of these G. Do not know

Do you know regarding the ADR reporting form? Yes No

How many sections are present in the ADR reporting form to validate ICSR (individual case safety report)? A. 1 B. 2 C. 3 D. 4 E. Do not know

What type of ADR should be reported? A. Known ADR B. Unknown ADR C. Serious/non-serious ADR D. All of these E. Do not know

ADR seriousness criteria include: A. Death B. Hospitalisation C. Disability D. Congenital anomaly E. Life-threatening F. Required intervention to prevent permanent damage G. All of these H. Do not know

Do you know about PvPI? Yes No

Which of the following challenges occur in implementing PvPI? A. Political pressure B. Lack of trained personal C. Underreporting culture D. Inadequate communication E. All of these F. Do not know

Which of the following is the WHO online database for reporting ADRs? A. ADR advisory committee B. Medsafe C. VigiBase D. MedWatch E. Do not know

Did you find the video helpful? Yes No

Will you share this knowledge regarding ADR reporting to the community? Yes No
